# Supplementary material for: Anisotropic and Highly Sensitive Flexible Strain Sensors Based on Carbon Nanotubes and Iron Nanowires for Human–Computer Interaction Systems
Source: Int J Mol Sci. 2023 Aug 22;24(17):13029. doi: 10.3390/ijms241713029 (PMC10488179; doi:10.3390/ijms241713029)
Supplement: Supplementary file 1 [file ijms-24-13029-s001.zip › supplementary.pdf]

Supplementary Materials: Video S1, Video S2, Video S3.

Figure S1, Figure S2, Figure S2

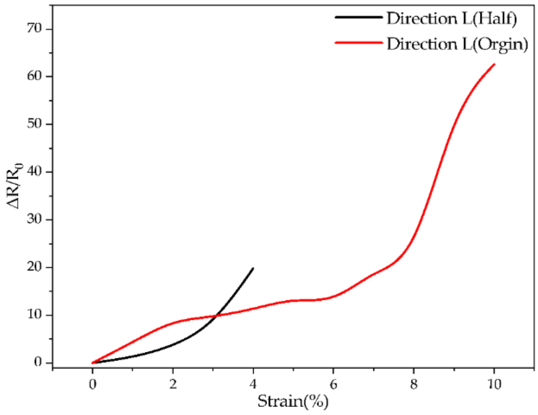

Figure S1. Strain testing of sprayed half-sensitive materials

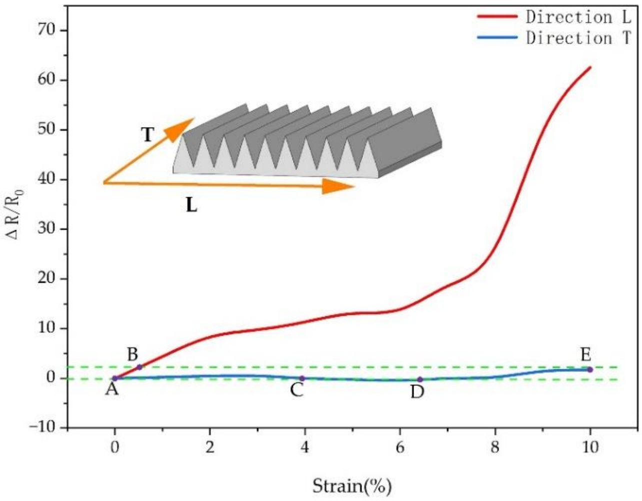

Figure S2. Change in $\Delta R/R_0$ value for directions T and L 10% strain

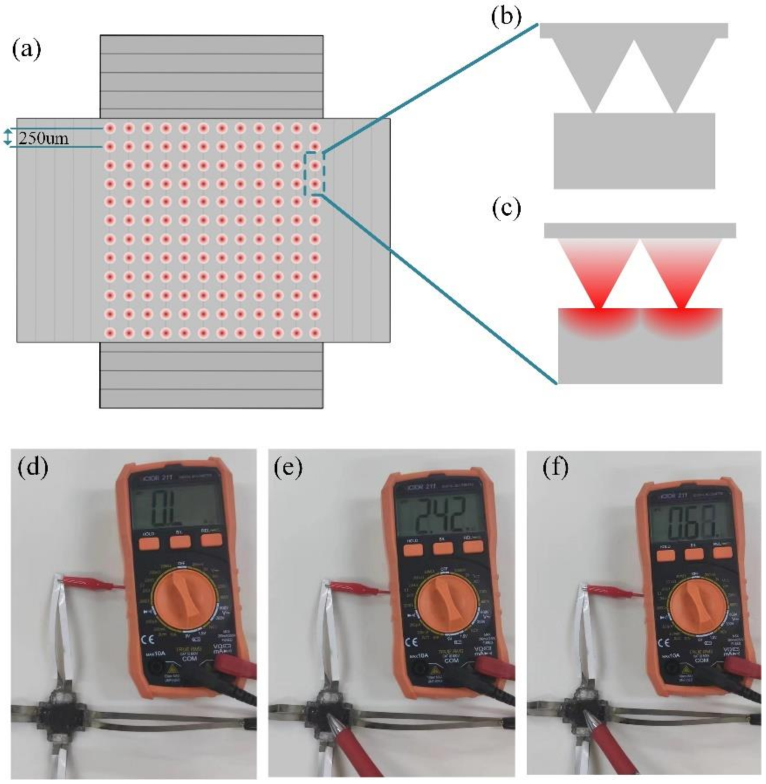

Figure S3. (a) Sensor pressure pixel detection schematic. (b)(c) Schematic diagram of the principle of action of pressure detection(d)(e)(f) Change in resistance value as sensor pressure increases
